# Integrating space, time, and culture in animal conservation practice

**DOI:** 10.1093/beheco/araf122

**Published:** 2025-10-31

**Authors:** William K Oestreich, Dawn R Barlow, Taylor A Hersh

**Affiliations:** Monterey Bay Aquarium Research Institute, 7700 Sandholdt Road, Moss Landing, CA 95039, United States; Department of Evolutionary Biology and Environmental Studies, University of Zurich, Winterthurerstrasse 190, 8057 Zurich, Switzerland; Marine Mammal Institute, Oregon State University, 2030 SE Marine Science Drive, Newport, OR 97365, United States; Marine Mammal Institute, Oregon State University, 2030 SE Marine Science Drive, Newport, OR 97365, United States; School of Biological Sciences, University of Bristol, 24 Tyndall Avenue, Bristol BS8 1TQ, United Kingdom

**Keywords:** animal culture, behavioral ecology, cetaceans, conservation, dynamic management, social learning

## Abstract

Recent theoretical integration of the spatiotemporal and cultural elements of animal behavior has led to increasing calls to incorporate animal culture into conservation. Implementation of this idea remains sparse due to disconnects between the theoretical concept of considering culture in animal conservation and the spatiotemporal approaches typically employed in conservation practice. Here we propose that this gap can be bridged by (1) clarifying that spatiotemporal conservation interventions inherently interact with culture regardless of whether this connection is acknowledged; and (2) strategically considering feasible “entry points” for considering animal culture in conservation practice. Recent advances in dynamic management strategies indicate the capacity for modern conservation approaches to integrate additional dimensions of animal behavior, and could serve as a particularly fruitful space for considering culture. Drawing on instructive examples from cetaceans, we examine instances where protection in space and time can facilitate the conservation of culture, and where focusing on conserving culturally distinct groups can yield protection in space and time. Human interventions that explicitly consider these interwoven dimensions in practice are achievable and can enable more holistic protections for diverse taxa.

## Teaser

Recent theoretical integration of the spatiotemporal and cultural elements of animal behavior has led to increasing calls to incorporate animal culture into conservation, yet implementation remains sparse. Here, we illustrate how this research-management gap can be bridged by (1) clarifying that spatiotemporal conservation interventions inherently interact with culture regardless of whether this connection is acknowledged; and (2) strategically considering feasible “entry points” for considering animal culture in conservation practice.

Social interactions in space and time shape culture—the inheritance of behavioral traditions through social learning from others (Whiten 2021). In turn, culture influences behavior in space and time. This interplay is evident in human lives and societies, and is increasingly recognized in non-human animals (Laland and Janik 2006). Behavioral ecologists are now establishing the inherent connections between culture and animal behavior in space and time (Brakes et al. 2019), fostering theoretical progress toward understanding how both spatiotemporal and cultural elements of behavior influence individuals' fitness and population dynamics. These developments have led to calls for translating theoretical understanding of animal culture into conservation applications (Carvalho et al. 2022; Greggor et al. 2025; Izar et al. 2025; Whitehead 2010). While the importance of conserving cultural units is theoretically attractive it has remained practically nebulous (but see Whitehead et al. 2023), with conservation efforts remaining predominantly focused on spatial management interventions and/or designating populations or stocks to conserve.

Here, we posit that the gap between animal culture research and conservation application can be bridged by providing the key contributing communities—animal culture researchers and conservation practitioners—with feasible points of entry to weaving together the dimensions of space, time, and culture in animal behavior and conservation. From the conservation practice point of view, this effort requires acknowledging that geographic conservation interventions inherently interact with cultural elements of animal behavior, and that leveraging these interactions can be valuable for achieving and improving desired management outcomes. From the animal culture research perspective, areas of conservation practice that have demonstrated the capacity to incorporate and benefit from considering additional dimensions [eg, dynamic management (DM); Maxwell et al. 2015] represent a promising avenue for injecting cultural considerations.

We highlight the feasibility of these approaches for folding knowledge of animal culture into conservation practice by drawing on illustrative examples from cetaceans (whales, dolphins, and porpoises). Cetaceans have provided repeated discoveries of social learning and culture across species and behaviors. Many populations exhibit long-range movement and communication behaviors which highlight the interactions among space, time, and culture, as well as their combined effect on sociality. Further, many cetaceans are highly mobile, cryptic, and/or long-lived, creating conservation challenges. Perhaps most importantly, despite many compelling examples of culture in cetaceans, practical implementation of this theoretical knowledge remains scarce (Brakes et al. 2025; Eguiguren et al. 2025; Garland et al. 2025). Cetaceans thus provide relatively well-studied, instructive examples on the intersections among space, time, and culture in behavioral ecology and their potential integration in conservation practice, yielding insights which can be adapted for a variety of species.

## Space, time, and culture are inherently interwoven in both behavior and conservation

**Fig. 1.**
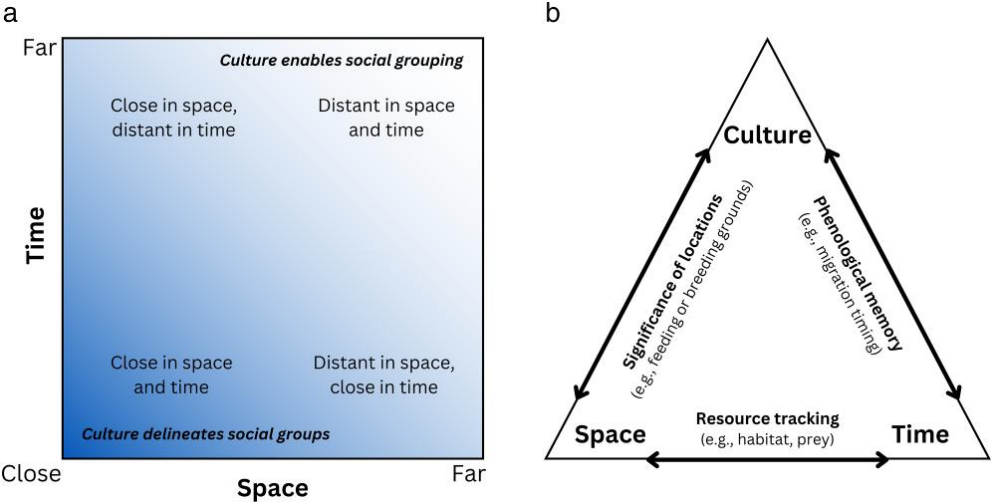
Conceptual schematics illustrating the intersecting dimensions of space, time, and culture in animal behavior. a) The interacting spatial and temporal dimensions of animal sociality. Shading indicates the likelihood of social grouping occurring; the presence of a cultural dimension enables delineation of distinct social groups near the origin (ie, when individuals are close in space and time), but also social grouping at the “far” ends of the axes (ie, when there is a disconnect in space and/or time). b) Depiction of the interacting effects of space, time, and culture, with examples of how the interactions can affect behavior.

The spatiotemporal and social dimensions of behavioral ecology are intrinsically intertwined (Webber et al. 2023). Social interactions most commonly occur between conspecifics that are proximate in space and time, though many animals have also evolved the capacity to transmit and acquire non-local social information (Fig. 1a). For example, acoustic signals (particularly in aquatic ecosystems) can propagate widely beyond the producing individual's proximate surroundings, enabling long-range and inconspicuous sociality in space and time (Tyack 2022; Dodson et al. 2024).

In some cases, these proximate or distant social interactions give rise to culture. Such socially learned and group-typical patterns are found in a diversity of behaviors, including foraging (Aplin et al. 2015), migration (Aikens et al. 2022), acoustic communication (Garland et al. 2011), mating site preferences (Warner 1988), and more. These cultural elements of behavior both influence and are influenced by spatial and temporal patterns of behavior (Fig. 1b), as social learning and cultural transmission often occur in specific places (eg, shared roosts) and at particular times (eg, breeding season). Culture also influences behavior in space and time. For example, socially transmitted information can lead to the emergence of culture around both spatial (Berdahl et al. 2018) and temporal (Oestreich et al. 2022) patterns of migratory behavior. Because of these links between space, time, and culture in behavior, spatiotemporal conservation interventions often have cultural ramifications—acknowledging and leveraging these connections can help clarify the relevance of culture in conservation practice.

Cetaceans provide useful examples in which social learning of a behavior is tied to a particular space and/or time, clearly illustrating the role spatial management can have in protecting cultural transmission of behavior. In the Gulf of Maine, humpback whales (*Megaptera novaeangliae*) exhibit a specialized, socially learned feeding behavior (“lobtail feeding”) which is culturally transmitted on their foraging grounds (Allen et al. 2013). Stellwagen Bank National Marine Sanctuary (a spatial conservation intervention) protects this culturally significant location and time (foraging grounds and season). In British Columbia, Canada, northern resident killer whales (*Orcinus orca*) rely on specific shallow gravel shorelines for “beach rubbing,” a culturally transmitted behavior in which they rub their bodies on the benthos at high tide (Williams et al. 2009). These smooth pebble beaches which killer whales use for beach rubbing are also protected by a spatial conservation intervention, the no-entry Robson Bight Ecological Reserve (Williams et al. 2009).

In other cases, cultural elements of behavior are not inherently tied to a specific place or time, but instead influence behavior over a broad range of spatiotemporal scales. For example, sperm whales (*Physeter macrocephalus*) produce socially learned vocalizations (codas), and different cultural groups of whales (vocal clans) exhibit preferences for specific coda types. These culture-specific coda preferences can be spread over ocean basins (ie, vocal clans span beyond overlap in space and time) and also delineate distinct social groups in sympatry (ie, vocal clans persist when overlapping in space and time) (Hersh et al. 2022). These discoveries have led to proposals for vocal clans to be treated as the unit of management for sperm whales (Brakes et al. 2019, 2021), rather than geographically or genetically defined stocks. Given that clan-level differences in behavior can be extreme (Vachon et al. 2022), conservation interventions targeting specific culturally defined vocal clans thus could have far-reaching ramifications in space and time. Cultural memory of migratory routes and destinations in southern right whales (*Eubalaena australis*) represents another case of how culture drives behavior and can influence protection in space and time. This species exhibits cultural traditions in migratory destination fidelity (Carroll et al. 2015), meaning that the loss of culturally distinct population segments has altered the places and times that represent critical habitat for this population (Harcourt et al. 2019). To be effective, protections at specific places and times must account for this cultural driver of behavior.

While documented cases of social learning and cultural transmission of behavior are hardly confined to cetaceans, these examples demonstrate how space, time, and culture are inherently interwoven and must be treated as such in conservation interventions. From the conservation practice point of view, resources are limited and hesitance to prioritize animal culture at the expense of more “practical” approaches is justified (Carvalho et al. 2022). But this is not a zero-sum game: incorporating cultural considerations can enhance the efficacy of “traditional” spatiotemporal management interventions, and in many cases, animal culture already influences and/or is influenced by existing conservation practices.

## DM as a conduit for further integration of animal culture into conservation practice

Historically, conservation efforts have strongly emphasized spatial planning. Recent years have seen greater incorporation of the temporal dimension, as in DM practices that provide protection that shifts in space over time (Maxwell et al. 2015). These DM approaches, which already integrate 2 key axes of animal behavior (Fig. 1b), represent a promising point of entry for including the third dimension of animal culture where possible.

By holistically integrating space- and time-based interventions, DM is better suited to align scales of environmental variability, animal movement, and human uses than either type of intervention alone (Maxwell et al. 2015; Welch et al. 2019). The potential for DM to minimize regulatory impacts on human activities while maximizing efficacy of conserving a focal species has been demonstrated (Hausner et al. 2021). Despite initial challenges in implementing DM regulations, a growing body of available knowledge, monitoring technologies, and predictive modeling tools (Payne et al. 2017) have enabled implementation of a growing number of DM programs (Oestreich et al. 2020 ), including notable examples from cetacean conservation. For critically endangered North Atlantic right whales that rely on highly urbanized coastal regions, acoustic and visual whale detections trigger dynamic vessel speed restrictions to minimize the risk of lethal ship strikes (Crowe et al. 2023). In Glacier Bay, Alaska, humpback whale surveys dictate the dynamic designation of “whale waters” where cruise ships must limit their speed (Harris et al. 2012). Off the West Coast of the United States, forecasts and observations of blue, fin, and humpback whale distributions provide near-real-time information to mariners to reduce the risk of ship strikes around major shipping ports (Abrahms et al. 2019).

To our knowledge, no existing DM plan explicitly incorporates animal culture, but we posit that DM can serve as an entry point for conservation practitioners to tangibly implement the theory behind cultural conservation. Existing DM plans already integrate spatial and temporal dimensions, triggering dynamic regulations based on species-level detections or forecasts. With sufficient knowledge of cultural units within a species, DM could be modified to be culturally specific. While integrating spatial, temporal, and cultural information into management practice creates challenges, this concept is not functionally different from many longstanding fishing or hunting regulations, where a specific demographic is allowed to be harvested only over a restricted area and time.

Cetaceans again provide cases particularly well-suited to this approach. Culturally distinct communities of killer whales (ecotypes) can be distinguished acoustically (Leu et al. 2022). Coupled with DM tools (eg, movement forecasts; Lin et al. 2025), this discernability paves the way for incorporating acoustic cultural identity into regional DM plans. For example, killer whales off Washington, United States are protected under the U.S. Marine Mammal Protection Act, but different ecotypes in the region are exhibiting vastly different population trajectories (Williams et al. 2024). As of January 2025, vessel operators must stay at least 1,000 yards away from Southern resident killer whales, which are critically endangered; in contrast, the exclusion zone around other sympatric cultural groups with better conservation outlooks is 200 yards (Washington State Legislature 2025). In this way, cultural identity is already being used to inform and enhance mitigation of human impacts dynamically in space and time. Sperm whales are another excellent candidate for culturally cognizant DM (Brakes et al. 2019, 2021), given that this species' behavioral ecology reduces the efficacy of static management areas (Eguiguren et al. 2025 ), cultural groups can be acoustically distinguished (Rendell and Whitehead 2003), and geographically sympatric cultural groups can have different population trajectories (Whitehead and Rendell 2004). Humpback and gray (*Eschrichtius robustus*) whales form distinct “migratory herds”—groups of whales unified by migration between common feeding and wintering grounds, learned and maintained through cultural memory (Martien et al. 2023). If herd-specific critical habitat can be understood and dynamically predicted in space and time, DM plans targeting these species could generate positive downstream cultural benefits by preserving cultural memory.

## Bridging the research-management gap to integrate across spatial, temporal, and cultural dimensions

Bridging the research-management gap requires consideration of the most strategic ways for each of these key contributing communities to reach out across this divide. From the viewpoint of conservation practitioners, focusing on culturally cognizant management actions might seem impractical in many cases (Carvalho et al. 2022) until we acknowledge that existing spatiotemporal approaches already influence culture and vice versa, and thus leveraging understanding of cultural transmission in behavior can enhance their efficacy. For academic researchers advocating for consideration of animal culture in conservation, it will be useful to build upon management tactics (eg, DM) that have the capacity to incorporate additional dimensions of animal behavior to enhance the efficacy of protections. DM itself required decades to move from theoretical recognition of its benefits (Hyrenbach et al. 2000) to successful implementation, a transition that has been accelerating (Oestreich et al. 2020) since foundational conceptual pieces clarified the key ingredients and communities enabling relatively rare (at the time) existing DM programs (Lewison et al. 2015; Maxwell et al. 2015). Integration of animal culture into conservation practice is perhaps at an earlier stage of a similar progression. We posit that there are feasible points of entry for research and management to find common ground; in doing so, these communities can enhance the efficacy of conservation interventions through incorporating knowledge about non-human animal culture.

## Data Availability

No original data are included in this manuscript.
